# Causality in cancer research: a journey through models in molecular epidemiology and their philosophical interpretation

**DOI:** 10.1186/s12982-017-0061-7

**Published:** 2017-06-07

**Authors:** Paolo Vineis, Phyllis Illari, Federica Russo

**Affiliations:** 1 MRC/PHE Centre for Environment and Health School of Public Health, Imperial College London, St Mary’s Campus - Norfolk Place, London, W2 1PG UK; 2 Department of Science and Technology Studies, University College, London, London, UK; 3 Department of Philosophy, University of Amsterdam, Amsterdam, The Netherlands

**Keywords:** Systems biology, Evidential pluralism, Information transmission, Difference-making, Mechanism

## Abstract

In the last decades, Systems Biology (including cancer research) has been driven by technology, statistical modelling and bioinformatics. In this paper we try to bring biological and philosophical thinking back. We thus aim at making different traditions of thought compatible: (a) causality in epidemiology and in philosophical theorizing—notably, the “sufficient-component-cause framework” and the “mark transmission” approach; (b) new acquisitions about disease pathogenesis, e.g. the “branched model” in cancer, and the role of biomarkers in this process; (c) the burgeoning of omics research, with a large number of “signals” and of associations that need to be interpreted. In the paper we summarize first the current views on carcinogenesis, and then explore the relevance of current philosophical interpretations of “cancer causes”. We try to offer a unifying framework to incorporate biomarkers and omic data into causal models, referring to a position called “evidential pluralism”. According to this view, causal reasoning is based on both “evidence of difference-making” (e.g. associations) and on “evidence of underlying biological mechanisms”. We conceptualize the way scientists detect and trace signals in terms of *information transmission*, which is a generalization of the mark transmission theory developed by philosopher Wesley Salmon. Our approach is capable of helping us conceptualize how heterogeneous factors such as micro and macro-biological and psycho-social—are causally linked. This is important not only to understand cancer etiology, but also to design public health policies that target the right *causal* factors at the macro-level.

**Electronic supplementary material:**

The online version of this article (doi:10.1186/s12982-017-0061-7) contains supplementary material, which is available to authorized users.

## Introduction

What we mean by “cause of a disease” has an obvious practical significance, for example for the development of drugs and preventive interventions (e.g. vaccination programmes). We believe that—building on current models of cancer causality, and in particular the model offered by “molecular epidemiology” [1]—there is the need to reconcile the conceptual interpretation of causality and its biological foundation. In this paper we address the meaning of causality in the case of cancer. For many cancers, causes are still elusive and there is confusion in the literature between cause and mechanism. Mechanisms do not need to be fully known for hazard identification (which can come from epidemiology alone, as was the case of smoking and cancer), but knowledge of mechanisms supports causal reasoning in both hazard identification and risk assessment (this is the idea of “evidential pluralism” that we also discuss later).

In addition to the practical implications, there are also important conceptual (philosophical) aspects in defining what a cause is, with cancer being an interesting case. This is particularly pressing, in the light of the advancements of molecular biology and the use of biomarkers in cancer research.

We first summarize the current views on carcinogenesis, and then explore the relevance of current philosophical interpretations of causality. We argue that using mechanisms to support causality claims in observational epidemiology is not just a matter of adding more fine-grained associations, but to understand “why” there are such associations. Our proposal is that the identification of causes of cancer rests on two components: (1) “difference-making”, and (2) “mechanism”. For example, in the recent controversy on the carcinogenicity of red meat [2], the epidemiological literature consistently detected an increase in risk of colon cancer among red meat eaters (difference-making), but further confirmation of a causal relationship came from the mechanisms involved, such as the formation of carcinogenic nitroso-compounds in the intestine of red meat eaters. Risk is just a measure of how much individual probability of cancer increases (e.g. in the exposed compared to the unexposed), conditionally on red meat consumption, but—with notable exceptions—a sound conclusion for a causal relationship also requires the identification of a plausible mechanism [3].

## The molecular basis of cancer: the microenvironment underlying macroenvironmental causes

We start with the mechanisms that underlie cancer onset, i.e. the sequence of molecular events that lead from a normal cell to a cancer cell. This is necessary to understand causality, in the framework of cancer as an evolutionary (Darwinian) process. It is important to stress that cancer is not a single entity, and therefore pathways leading to cancer onset are diversified. There have been several important developments in the molecular interpretation of carcinogenesis in recent decades, including (a) a wide set of mutagenic events which encompasses single base substitutions as well as larger structural genetic alterations; (b) an understanding of the crucial role of epigenetic changes (defined as functional changes in DNA that do not involve a change in the nucleotide sequence); (c) an acknowledgement of the importance of selection of mutated or epimutated cells; and (d) the unifying concept of “branched evolution”, i.e. evolution occurs in a branched manner in several tumor types, leading to intratumor diversity, with the selective advantage of any genotype depending on the environment [4].

There are several implications for primary prevention derived from this definition (represented in Additional file 1: Figure S1).

- Cancers occur in stages that correspond to increasing complexity of molecular changes (“intratumor diversity”), with two metastases or two areas in the same localized tumour having a different set of mutations.
- Mutations can be neutral, detrimental or favorable for the expansion of a cell clone, depending both on the micro-environment, that exerts a selective pressure, and the previous history of mutations in the same cell. The latter concept is called “historical contingency” [5] and corresponds to the influence that previous mutations have on the effects of subsequent mutations on protein structure and function, and also on the evolution of entire gene regulatory networks [5].
- In the onset of cancer in individuals, both mutagens and “selectogens” play a role, i.e. the individual cancer reflects the history of exposures that both induce mutations and facilitate the selection of existing mutations. Selectogens may include known risk factors for cancer, such as the metabolic syndrome, that are unlikely to have a mutational mechanism as their main mode of action, and may predominantly act by promoting the selection of cells already carrying somatic mutations.

Smith et al. [6] have identified ten “hallmarks of carcinogens”, in the context of the IARC Monographs (Table 1); these correspond to the main mechanisms identified so far in the pathways to cancer, and at least four of these are not based on mutagenesis, e.g. chronic inflammation. It is likely that in the “branched evolution” paradigm, risk factors acting via these mechanisms play the role of selectogens. It will also be critically important to understand how such non-mutagenic environmental exposures may interact with cellular processes that maintain the fidelity of DNA (e.g. DNA repair and replication), thus affecting the “endogenous” mutations seen in different types of human tumours.

**Table 1:** Key characteristics of carcinogens (from Smith et al. [6])

1. Is electrophilic or can be metabolically activated
Parent compound or metabolite with an electrophilic structure (e.g. epoxide, quinone, etc.), formation of DNA and protein adducts
2. Is genotoxic
DNA damage (DNA strand breaks, DNA protein cross-links, unscheduled DNA synthesis), intercalation, gene mutations, cytogenetic changes (e.g. chromosome aberrations, micronuclei)
3. Alters DNA repair or causes genomic instability
Alterations of DNA replication or repair (e.g. topoisomerase II, base-excision or double-strand break repair)
4. Induces epigenetic alterations
DNA methylation, histone modification, microRNA expression
5. Induces oxidative stress
Oxygen radicals, oxidative stress, oxidative damage to macromolecules (e.g. DNA, lipids)
6. Induces chronic inflammation
Elevated white blood cells, myeloperoxidase activity, altered cytokine and/or chemokine production
7. Is immunosuppressive
Decreased immunosurveillance, immune system dysfunction
8. Modulates receptor-mediated effects
Receptor in/activation (e.g. ER, PPAR, AhR) or modulation of exogenous ligands (including hormones)
9. Causes immortalization
Inhibition of senescence, cell transformation
10. Alters cell proliferation, cell death or nutrient supply
Increased proliferation, decreased apoptosis, changes in growth factors, energetics and signaling pathways related to cellular replication or cell cycle

## Macroenvironmental causes of cancer

How are these concepts, at the level of the micro-environment, connected to external exposures in the macro-environment? Based on epidemiological evidence, we know that some 40–50% of cancers would be preventable if current knowledge about risk factors were to be translated into preventive interventions [7–9]. There is broad consensus on these estimates in the epidemiological community, though the concept of “attributable risk” is still debated and is methodologically weak (for limitations see [10]).

These preventable cancers are for the most part explained by external (or internal—such as endogenous nitrosation) exposures that are unlikely to act in isolation: even a “necessary” cause of cancer, human papilloma virus (HPV), is itself not sufficient to cause cervical cancer in an individual. Though all cervical cancers need exposure to HPV, being exposed to HPV needs other additional components in the causal constellation that led to an individual’s cancer. On a population scale, HPV is probably able to explain 100% of cervical cancer cases (in principle cervical cancer can be eradicated by vaccination), but each individual case is not entirely explained by HPV alone: for example, exposure to the virus happens in a socio-economic context that is also part of the etiology of cancer (including other sexually-transmitted infections and behaviours that interact with the virus).

The model of causation that applies to single individuals is called the “sufficient-component-cause framework”, and it considers sets of actions, events, or states of nature that together lead to the outcome under consideration. This concept has been popularized by Rothman et al. [11] through the metaphor of “pies”: the constellation of exposures that has led to cancer in an individual or a group of individuals is represented as a pie where the slices are different components and the totality of them is causally sufficient. The model gives an account of the multiple causes that in their combination lead to a particular effect. The model usefully captures multi-causality and the interaction between component causes (in other words their “organization”).

The above concepts allow us to bring together two domains that have been separated so far: the “ecology of cancer” at a population level (the macro-environment) and the mechanisms of carcinogenesis (the micro-environment) at the individual level. Additional file 2: Figure S2 shows the “ecology” of some common cancers in different countries, though the picture is rapidly changing because of globalization [12]: the Figure suggests that in each area there are some forms of cancer that prevail due to the local predominant exposures. Such exposures are likely to be a mixture of mutagens, such as aflatoxin B1, and selectogens, such as chronic inflammation caused by the hepatitis B virus; these two factors combine to increase the risk of e.g. hepatocellular carcinoma in Asia and sub-Saharan Africa. In other cases a single complex mixture, e.g. tobacco smoke, can comprise a combination of mutagens and selectogens.

The future challenge will be to monitor this complex and changing ecology of cancer (and other non-communicable diseases), and to relate these changes and interpret their effects with respect to the micro-environmental modifications. Equally, starting with the molecular modifications observed at the level of the micro-environment can reveal clues as to the ecology of cancer at the macro-environmental level. An example comes from the recent observation that renal cell cancers in some regions in Europe have a somatic mutation spectrum that reflects exposure to an environmental carcinogen, aristolochic acid, previously considered as a risk factor for upper urothelial tract cancers [13].

The attempt to connect the external (macro) with the internal (micro) environment has been explored within “exposome” research [14]. While the macro-environment represents the “external exposome”, the micro-environment can be explored as a part of the “internal exposome” using the new high-throughput technologies of epigenomics, transcriptomics, miRNA, proteomics and metabolomics. The connection between the external environment and internal biological changes has been the goal of molecular epidemiology for decades, as expressed for example in Schulte and Perera’s [1] book. New technologies can in principle allow us to monitor how the micro-environment can lead to selection of mutations and thus identify selectogens as additional targets for prevention. There are great expectations towards these omic technologies for the development and validation of a suite of new biomarkers to monitor the micro-environmental changes underlying cancer development.

It is becoming increasingly clear that non-communicable diseases are influenced by events that took place throughout an individual’s life-course, in both the macro- and micro-environments. The concept of “branched evolution” stimulates fresh thought on the relevance of timing of exposures in relation to subsequent cancer risk. For example, given that certain “driver” mutations may only exert their carcinogenic effects in the context of favorable selective conditions at the level of the micro-environment, one can postulate that past exposures may leave genetic or epigenetic alterations that are only expressed far later in time, contingent on subsequent exposures. The fact that adult diseases such as cardiovascular diseases or cancer were influenced by previous exposure including in utero, e.g. nutrient deficiency in later generations due to the Dutch famine during the World War II [15], suggests that the whole lifecourse has an impact on adult disease. This poses particular challenges to the identification of risk factors that may exert a type of “hit-and-run” effect.

In sum, the most recent understanding of cancer etiology presents us with a complex scenario where disease (here, cancer) is the result of a process in which factors in the micro- and in the macro-environment interact. Such interactions are consistently found in the associations identified by studies in molecular epidemiology. The challenge for molecular epidemiology is therefore to explain how biological mechanisms across the micro- and macro-environment contribute to causal reasoning.

## A philosophical understanding of cancer etiology

### Biomarkers: the link between the macro- and the micro-environment

In order to causally link the micro- and macro-environments, omic technologies provide a key set of instruments in cancer research: these allow us to connect exposure and disease by finding the “right” biomarkers. Biomarkers are key in causal analysis in cancer research and play a major role in our conceptualization of cancer causation. This is well expressed in the diagram that connects exposure markers, early effect markers and susceptibility markers in the classical “molecular epidemiology” paradigm, as described in Schulte and Perera’s [1] book and further elaborated recently [16].

In 1998, the National Institute of Health Biomarkers Definitions Working Group defined a biomarker as “a characteristic that is objectively measured and evaluated as an indicator of normal biological processes, pathogenic processes, or pharmacologic responses to a therapeutic intervention.” Biomarkers are largely *constructed* by cross-checking data that are generated by some machines (e.g. mass-spectrometry) and subsequently analyzed using other machines (computers and their algorithms). An important question therefore concerns the kind of *ontological status* that we should give to biomarkers. Strictly speaking, they don’t seem to be just ‘objects out there’. Schulte and Perera [1] describe biomarkers in terms of ‘events’ in the continuum from exposure to disease. But even within this continuum, such markers may represent a genuine event (e.g. direct exposure to a pollutant), may be correlated with such an event (the classical example of yellow fingers in heavy smokers), or even be a predictor of the event without being causally associated to it (like the association between two X chromosomes and the propensity to wear skirts). The fact that biomarkers are hardly corresponding to “causal” molecular entities does not imply that they cannot be measured. In fact, this is what molecular epidemiology routinely does. But, as Schulte noticed as early as 1993, there are multiple ways of defining and measuring biomarkers, which raises the question of their ontological status.

The issue gets even more complex because molecular epidemiology is not interested in finding biomarkers *per se*, but in understanding the *continuum* of disease development from early exposures, via finding biomarkers. Similarly, in other contributions, the technologies used to detect biomarkers (some of which are called omic technologies) are said to provide the ‘missing link’ between exposure and disease or, given the previous discussion, between the macro- and the micro-environment [17–19].

This conceptualization of biomarkers search—i.e. as the continuum linking exposure and disease—emphasizes *processes* rather than *things* or *objects*. This calls for two remarks. On the one hand, biomarkers are not entities, things to which we can attribute some causal power, in the same sense as HPV virus has the power to initiate the onset of cervical cancer. Instead, biomarkers are clues, indicators, *markers* to detect in order to reconstruct the missing link. On the other hand, and related to the previous point, we need to say in which sense, if any, these continuous links, or processes, between exposure and disease are *causal*. This is all the more important because we seek to link *heterogeneous* levels as the macro- and the micro-environment. In sum, our approach aims to address two main questions: first, how to understand causal *production* from the macro- to the micro-environment, and second, why it is important to have a coherent conceptualization of such causal links. We discuss these two issues in reverse order: spelling out the second question will provide further motivation for our approach.

### Information transmission and the link between macro- and micro-environment

Finding a coherent conceptualization of the link between the macro- and the micro-environment is important for the following reason. The macro-environment consists of biological agents, pollutants and chemicals we are exposed to, but also of social interactions and “psycho-social factors”. The micro-environment, instead, is made of biochemical and molecular processes measured at different “omic levels”. How to make the causal link between the macro- and the micro-environment plausible, beyond a “coarse-grained” difference-making relation between the two?

By and large, traditional epidemiology has done this successfully for a long time: establishing robust associations between classes of exposures and classes of diseases. But with the advent of molecular epidemiology, these associations also relate factors at very different levels (the micro and macro environments). This rests on a *change of the scale of measurement*: environmental exposure has traditionally been assessed by measuring the *levels* of individual chemicals in, say, air or water. Thus newer finer-grained measurements initially try to restore some kind of “scale homogeneity”: measure the level of a pollutant or of a chemical externally and then measure changes at genomic, transcriptomic, proteomic, or metabolomic levels internally. Although ‘scale homogeneity’ is restored through making all measurements chemico-biological measurements, the problem is not solved.

In fact, measurements now taking place at the same level allow the researcher merely to establish another association or series of associations (difference-making relations), albeit at a much lower level now. For instance, we might establish a robust correlation between the level of a certain chemical in the air and the biomarker of early clinical changes of a targeted disease (lung cancer). But this doesn’t establish a *causal link* yet. It only estimates a more precise measure connecting levels of hazards and levels of omic changes. On the one hand, to establish a causal link we still need to find the right “intermediate” biomarkers, the ones that are linked to exposure and to disease. To be sure, this search (finding appropriate biomarkers) obviously relies upon studying associations, e.g. via omics analyses. On the other hand, we need to place this reconstructed link into a plausible network of relations (i.e. the mechanisms of carcinogenesis described in the first part of the paper), and this is precisely the kind of ‘biological thinking’ mentioned earlier. It is important to note that linking, here, cannot be seen by the naked eye, and not even using sophisticated experimental set-ups. Instead, the scientist reconstructs the linking by putting together the pieces of the *evidential puzzle*, just as a crossword puzzle [20]. Biological theory needs to be complemented with the results of omic analyses, which in turn need sophisticated and complex statistical analyses. It is in this sense that cancer etiology needs a *plurality of evidence* from which to make causal inferences. All this requires considerable empirical evidence and much *interpretation* of the evidence using the appropriate concepts. One such concept is *information transmission,* as we argue later.

A second, more important, reason why the problem is not solved is that although homogeneity in the scale of measurement is restored by using biological measurements, this makes the results harder to interpret, because the interpretation still has to identify causes at the macro level, i.e. the level of environmental exposure causing disease. We need this causal knowledge to design appropriate public health interventions. To sum up: we measure everything at the micro-level (level of pollutant, and level of metabolite) but ultimately what we want to know is how and to what extent environmental pollutants or psycho-social factors cause diseases. The problem molecular epidemiology faces is: how can we understand macro-factors causing micro-factors, or vice versa? What we have to establish is a continuous linking, not just (finer-grained) correlations at a different level of measurement. Continuous linking can be conceptualized as information transmission, as we explain next.

### Productive causality as information transmission

We mentioned earlier that causal claims about exposure and cancer involve statements about risks, i.e. *difference*-*making*: whether certain exposures are good predictors of disease, at different stages of disease development, or at different stages of life, etc. Simultaneously, we also look for evidence about *how* exposure leads to developing disease. Typically, ‘how’ exposure leads to disease has been understood in terms of the mechanisms that produce disease, mainly with the study of biomarkers. Mechanisms provide us with information about how causes *produce* effects. This position is called, in philosophy, *evidential pluralism*, to emphasize the need for multifold (or multi-layered) evidence in order to establish causal claims [3]. A prestigious example of evidential pluralism is the joint use of epidemiological evidence (difference-making) and mechanistic evidence (productive causality) in the Monographs of the International Agency for Research on Cancer [21].

The difference-making component of evidential pluralisms is, in a sense, less controversial than the productive component, as even theorizers of agnostic data-driven approaches will agree that the search for robust statistical associations lies at the very heart of data-intensive science. What remains controversial is what biomarkers are marks *of* within a mechanistic understanding of cancer etiology. This is problematic because, as discussed before, we want to establish links between macro- and micro-factors. On the one hand, causal relations are not reduced to bio-chemical relations and, on the other hand, they are not mere (finer-grained) statistical associations among macro-variables.

If the causal link connects factors at different scales and of different types, then the notion of productive causality (i.e. how causes and effects are linked) needs reconceptualization. But the type of linking sought may be different depending on the scientific context or the purpose of the causal question.

There are several candidates for characterizing links; we mention the two most prominent here. First: Wesley Salmon’s “mark transmission theory” [22–25]. In Salmon’s view, the central question is how to distinguish between *causal* processes and non-causal (or pseudo) processes. Simply put, causal process transmit marks, while pseudo-processes don’t. Think about what happens when introducing a mark in a process: if the process is causal, the mark persists at a later stage. A stock example is denting a car, and observing that the dent is transmitted along with the movement of the car, while its *shadow* will not further transmit the mark. This shows that the moving car is a causal process, while a moving shadow is not. However, not every process can be marked, and Salmon formulated the approach in counterfactual terms: a casual process is one that *could* be marked and that *could* transmit the mark. Causal processes, in this approach, are those transmitting physics quantities, such as energy or momentum (think of billiard balls colliding). However, this approach is tailored to physics and does not provide the conceptual tools to understand the macro–micro linking mentioned above. Second: the ‘complex-systems’ approach [26]. According to this approach, to establish causal relations one needs to identify mechanisms, in the sense of complex systems that link causes and effects. Such approach, however, emphasizes the *organization* of different components of a mechanism, rather than the *continuum* linking exposure to disease. For instance, a mechanistic explanation sheds light on the way a gene normally is methylated, and how it is hypomethylated when exposed to tobacco smoking. We can shed light on these mechanistic aspects by identifying the relevant molecular entities and activities involved, and their *organization*. But this is not very illuminating about the continuous link between exposure and disease, that is the *process* initiated with exposure and that eventually leads to disease development, via several intermediate stages.

The link is instead better conceptualized using the notion of “information transmission”. Note that information transmission does not coincide with transfer of biological information between macro- and micro-factors. Instead, information transmission refers to how the scientist reconstructs the linking between macro- and micro-factors, putting together all the available pieces of the evidential puzzle. In other words, information transmission is at the level of epistemology, not of ontology.

In a previous article [27] we suggest that we need to explore the prospects of the notion of information that comes from the way scientists themselves explain the role of biomarkers; in this context, the idea of ‘picking up signals’ recurs, for instance:

From these two parallel analyses [statistical analyses], we obtained lists of putative markers of (i) the disease outcome, and (ii) exposure. These were compared in a second step in order to identify possible intersecting signals, therefore defining potential intermediate biomarkers [28].

What is the *signal* that we have to pick up? In what sense will this give us the sought production-relation between exposure and disease? Our suggestion is to conceptualize the detection and tracing of signals in terms of *information transmission*, as sketched above. This, we submit, is a generalization of Salmon’s mark transmission theory [27].

The key difference with Salmon processes consists in the marking aspect. Salmon’s approach rests on the *introduction* of the mark. However, in most cancer research we look for *existing* marks from exposure to disease that transmit along the process, without introducing them ourselves. Cancer research is largely an observational rather than an experimental science.

This understanding of causal production as information transmission takes full advantage of a conceptualization in terms of mark transmission in processes, without being tied to the quantities of physics, say energy or momentum, being transmitted. It also takes full advantage of a conceptualization in terms of mechanisms, because knowledge of relevant molecular or biochemical mechanisms will indicate where to look for signals, for instance choosing appropriate omics levels for the analyses of biological specimens. In this sense we say that mechanisms are *information channels:* “biochemical or molecular spaces” where we look for the flow of information that we try to intercept using biomarkers [27].

Ultimately, we want to understand the whole phenomenon of carcinogenesis: all the relevant omics levels involved, how they interact, and build reliable models of the dynamic evolution of whole systems under many different exposure conditions. The concept giving the dynamic evolution is *information transmission*. The flow is in the link, and the link, as suggested, is best thought of as informational. More precisely, it is given by the *scientists’ reconstruction* of the information transmission through the different types of analyses, i.e. by putting together all the pieces of the “evidential puzzle”.

The question remains: what exactly does information mean? In Genome Wide Association Studies (GWAS), there is at least some possibility of a clear (univocal) definition of information, as genes are more clearly defined than in most omic measurements, and substantive informational concepts make sense when applied to genes. Instead, in Exposome Wide Association Studies (EWAS) information is still not well-defined [27]. (Often omic “signals” are only “features”, i.e. they need to be decoded after discovery). However, the diversity and richness of informational concepts (many of which currently being developed and discussed), is not a weakness of an informational approach, but a virtue. This is captured, for instance, by philosophical accounts, especially those developing *qualitative* notions of information. One such account is *semantic* information, namely what the observer (here, the scientist) can process, looking at the data, omic analysis, biological theory, etc. It is in this sense that information transmission cannot be reduced to biological information, but it is certainly part of it.

One advantage of information transmission is that it is capable of offering a structure for thinking about how heterogeneous factors such as micro and macro-biological and social—are linked; this is a pressing issue in the light of results of omic studies and also for the design of public health policies.

## Conclusions

Systems biology is driven by technology (the development of omics) and by statistical modelling and bioinformatics. It is high time to bring biological thinking back. To address the new challenges of epidemiology, the concept of the “exposome” has been proposed, initially by Wild et al. [14], and then expanded by others, particularly Rappaport and Smith [29] who functionalized the exposome in terms of chemical signals detectable in biospecimens. This is consistent (and in fact is an extension) of previous work on molecular epidemiology by e.g. Schulte and Perera [1]. The canonical exposome concept refers to the totality of exposures from a variety of sources including chemical agents, biological agents, radiation, and psychosocial components from conception onward, over a complete lifetime [24]. We offered a unifying framework to incorporate omic data into causal models, using the position called “evidential pluralism”: causal reasoning is based on both “difference-making” and the underlying biological mechanisms. In particular, we conceptualize the way scientists detect and trace signals in terms of *information transmission*, which is a generalization of Salmon’s mark transmission theory. One advantage of information transmission is that it is capable of helping us conceptualize how heterogeneous factors such as micro and macro-biological and psycho-social—are causally linked. What we want to make clear is that—though it is often thought that going down the molecular level means to add details to a macro-level causal relations—this is in fact not the case. A good example in this respect is epigenetics, which shows that the way in which the macro is causally linked to the micro is not simply a matter of adding details to the same mechanism, but a matter of transmission of information from outside the body downstream to DNA and then the informational chain in the cell. This is important not only to understand cancer etiology, but also for the design of public health policies. In fact, public health interventions cannot target biomarkers, but the right causal factors at the macro-level, such as environmental hazards and socio- economic and psychological factors.

## Additional files

**Additional file 1: Figure S1.** Branched evolution (3).

**Additional file 2: Figure S2.** The macro-environment: ecology of cancer in a historical perspective. Examples are purely illustrative.
