# Supplementary figures and images for: Causality in cancer research: a journey through models in molecular epidemiology and their philosophical interpretation
Source: Emerg Themes Epidemiol. 2017 Jun 7;14:7. doi: 10.1186/s12982-017-0061-7 (PMC5463386; doi:10.1186/s12982-017-0061-7)

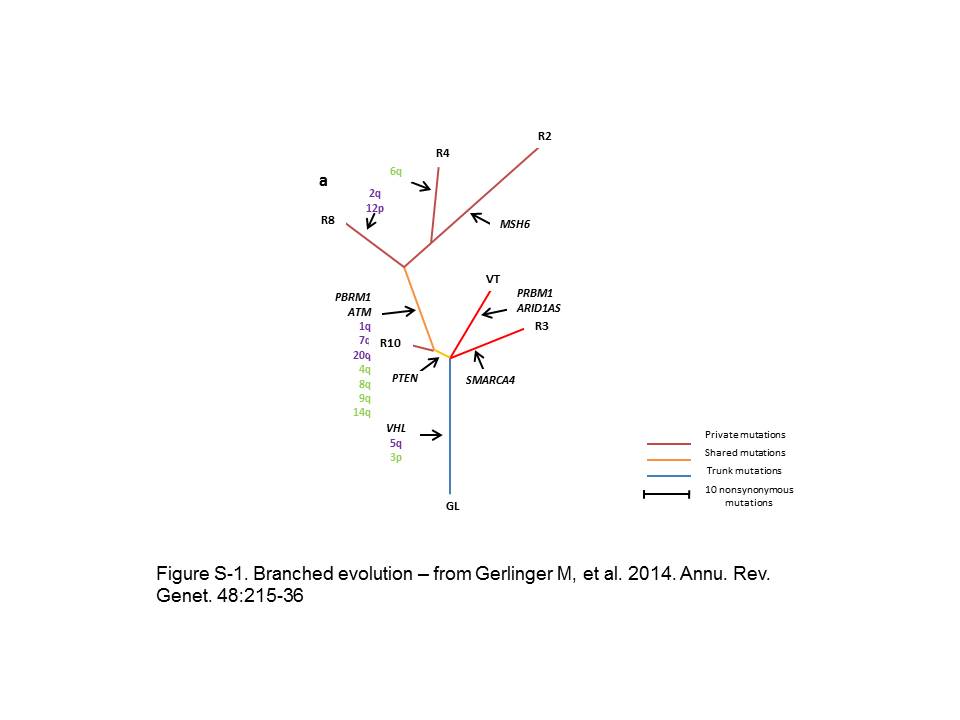

Supplement: Supplementary file 1 — Additional file 1: Figure S1. Branched evolution (3). [file 12982_2017_61_MOESM1_ESM.jpg]

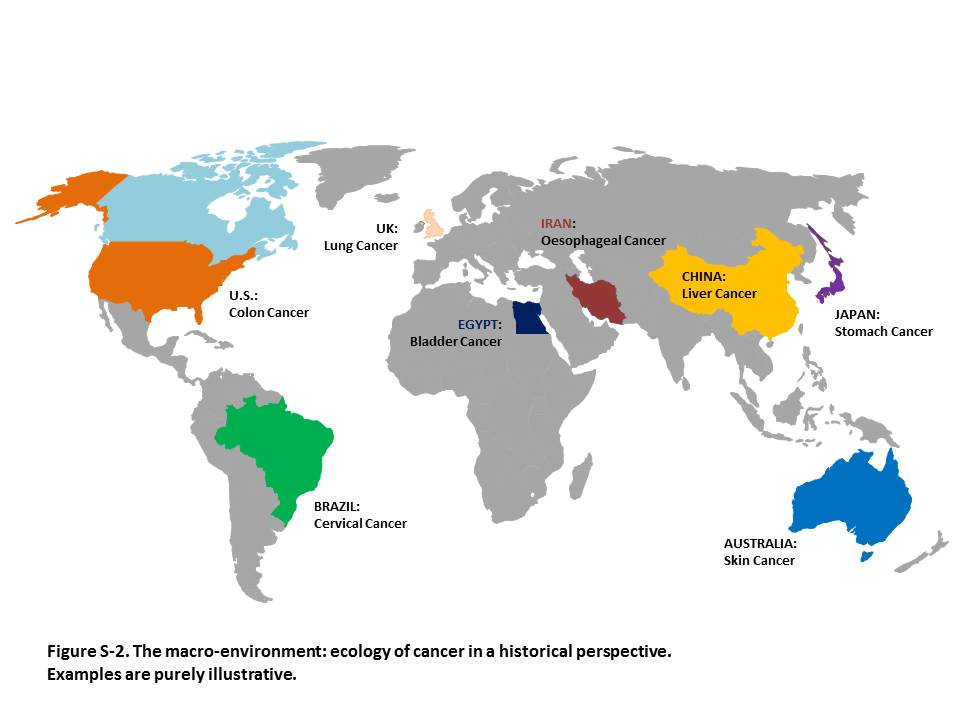

Supplement: Supplementary file 2 — Additional file 2: Figure S2. The macro-environment: ecology of cancer in a historical perspective. Examples are purely illustrative. [file 12982_2017_61_MOESM2_ESM.jpg]
